# Calcium influx-induced lytic cell death disrupts skin immune homeostasis

**DOI:** 10.1038/s41421-023-00623-2

**Published:** 2023-12-19

**Authors:** Yingxue Du, Xiangbing Qi, Lei Zhang, Yong Yang, Ting Chen

**Affiliations:** 1 National Institute of Biological Sciences, Beijing, China; 2 Peking University–Tsinghua University–National Institute of Biological Sciences Joint Graduate Program, School of Life Sciences, Tsinghua University, Beijing, China; 3 Tsinghua Institute of Multidisciplinary Biomedical Research, Tsinghua University, Beijing, China; 4 School of Biomedical Engineering, Capital Medical University, Beijing, China; 5 Chinese Institute for Brain Research, Beijing, China; 6 Institute of Dermatology, Chinese Academy of Medical Sciences and Peking Union Medical College, Nanjing, Jiangsu China

**Keywords:** Skin stem cells, Cell death, Immunology

Dear Editor,

Programmed lytic cell death is crucial in maintaining tissue homeostasis and contributing to pathogenesis. However, our understanding of whether calcium can trigger lytic cell death through calcium-permeable channels responding to environmental stimuli remains limited^1^. TRPV3, a calcium-permeable channel in the cell plasma membrane, is highly expressed in skin keratinocytes—the first line of defense against various physical and chemical environmental stimuli^2^. Here, we found a unique mechanism involving TRPV3-mediated calcium influx-induced lytic cell death in the skin, disrupting skin immune homeostasis.

Various stimuli, including heat, natural activators, and patient-specific gain-of-function mutations, can potentially activate TRPV3 (Fig. 1a)^3^. Upon subjecting TRPV3-expressing HeLa cells (Supplementary Fig. S1a, b) to the TRPV3-specific activator 2-aminoethoxydiphenyl borate (2APB) and camphor cocktail^4^, we observed membrane bubbling, nuclear condensation, phosphatidylserine externalization, and cell membrane rupture (Fig. 1b). Additionally, we measured two key indicators of lytic cell death: a decrease in ATP levels and the release of LDH from cells (Fig. 1c, d; Supplementary Fig. S1c, d). All these findings suggest that TRPV3 activation leads to lytic cell death. This lytic cell death phenomenon was also observed in cases of TRPV3 G568V gain-of-function mutation expression or heat activation (Supplementary Fig. S1e–g).

**Fig. 1:**
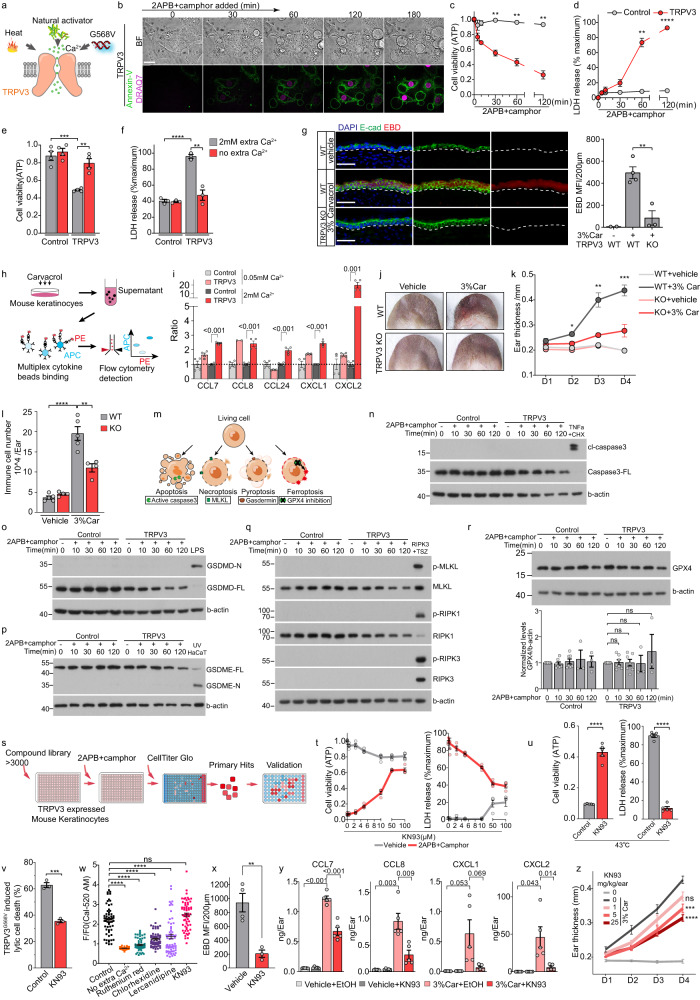
TRPV3 activation leads to extracellular calcium-dependent lytic cell death and disrupts skin immune homeostasis. **a** Schematic of TRPV3 activation. **b** Live images of HeLa cells expressing mTRPV3 treated with 2APB (100 μM) and camphor (2 mM) cocktail during the indicated time. BF bright fields. Annexin V (green) indicated phosphatidylserine externalization, and DRAQ7 (purple) presented cell membrane integrity disruption. Scale bar: 20 μm. **c**, **d** Cell viability (**c**) was assessed by cellular ATP level (*n* = 3), and membrane integrity (**d**) was measured by LDH release (*n* = 3). **e**, **f** Cell viability (*n* = 4, **e**) and membrane integrity (*n* = 3, **f**) of control or mTRPV3-expressing HeLa cells after 2APB and camphor cocktail treatment with or without extracellular calcium. **g** Evens blue dye (EBD, Red) co-staining with E-cad (green) of mouse ear section. Scale bar: 50 μm. **h** Schematic of multiple chemokines detection workflow. **i** Pro-inflammatory chemokines relative release. **j** Appearance of vehicle- and carvacrol-treated ears of *TRPV3* WT or KO mice at day 4. **k** Ear thickness of vehicle- and carvacrol-treated ears of *TRPV3* WT or KO mice (*n* ≥ 4). The statistical significance of the ear thickness between *TRPV3* WT and KO mice treated by carvacrol treatment was shown. **l** Total immune cell numbers per ear of vehicle- and carvacrol-treated *TRPV3* WT or KO mice measured by flow cytometry (*n* ≥ 4). **m** The well-known regulated cell death processes. **n**–**r** Western blot analyses of control or mTRPV3-expressing HeLa cells treated by 2APB and camphor with the indicated time. TNFα and CHX (cycloheximide) treated HeLa cells acted as cleavage caspase3 positive control. HeLa cells expressing RIPK3 stimulated by TNFα, Smac mimetic, and a pan-caspase inhibitor zVAD (TSZ) acted as RIPK1/RIPK3/MLKL phosphorylation positive control. Lipopolysaccharide (LPS) electroporation of HeLa cells served as cleavage GSDMD positive control. Ultraviolet radiation-treated HaCaT cells acted as cleavage GSDME positive control. **s** Workflow of the high-throughput chemical compound screen. **t** Cell viability (*n* ≥ 3) and membrane integrity (*n* ≥ 3) were measured in mTRPV3-expressing mouse keratinocytes with the indicated treatment. **u** Cell viability (*n* = 5) and membrane integrity (*n* = 5) were measured in HeLa cells expressing mTRPV3 cultured at 43 °C for 3 h with 50 μM KN93 or control. **v** Mouse TRPV3 G568V mutant-2A-H2BRFP was transfected to mouse keratinocytes for 14 h and cultured with 50 μM KN93 or control for 10 h. The percentages of DRAQ7-positive cells indicated as lytic death cells in total RFP-positive cells were recorded (*n* = 3). **w** Mouse keratinocytes expressing mTRPV3 were pre-treated with the indicated compounds before calcium imaging, and F/F0 per cell was recorded 5 min after 2APB and camphor treatment (*n* ≥ 30 cells, from independent experiments). **x** EBD MFI of ear section in carvacrol-induced dermatitis mice treated with KN93 or vehicle (*n* ≥ 3). **y** Pro-inflammatory chemokines levels in mouse ear after the indicated treatments on day 3. **z** Ear thickness of carvacrol-induced dermatitis mouse treated with KN93 (*n* ≥ 4). The statistical significance of the thickness between vehicle or indicated KN93 dosage treated ear on day 4 was shown. All the above data are presented as mean ± SEM.

2APB and camphor cocktail activates TRPV3, resulting in extracellular calcium-dependent calcium influx (Supplementary Fig. S1h). To investigate whether TRPV3 activation-induced lytic cell death is dependent on extracellular calcium, we treated control and TRPV3-expressing HeLa cells cultured in HBSS with or without extracellular calcium using 2APB and camphor cocktail. The removal of extracellular calcium restored lytic cell death to the control level (Fig. 1e, f). Since TRPV3 is primarily expressed in keratinocytes in the skin (Supplementary Fig. S1i–l), and its expression is significantly reduced in cultured mouse keratinocytes in vitro to nearly absent levels (Supplementary Fig. S1m), we established a stable mouse keratinocyte cell line expressing TRPV3. We also observed the TRPV3-mediated calcium influx-induced lytic cell death feature when TRPV3 was activated in mouse keratinocytes (Supplementary Fig. S1n–r).

To investigate whether TRPV3 activation induces lytic cell death in vivo, we applied the TRPV3 activator carvacrol topically to mice (Supplementary Fig. S1s). In vitro, we observed that TRPV3-expressing mouse keratinocytes undergo lytic cell death in a concentration-dependent manner upon exposure to carvacrol (Supplementary Fig. S1t). We quantified epidermal cell membrane rupture by measuring the fluorescence signal of the cell membrane-impermeable dye Evans blue in the epidermis. After carvacrol treatment, a positive signal was evident in the epidermis of wild-type (WT) mouse ears but not in *TRPV3* knockout (KO) mouse ears (Fig. 1g). These findings demonstrate that TRPV3 activation leads to lytic cell death both in vitro and in vivo.

Next, we sought to understand the consequences of TRPV3 activation-induced lytic cell death. TRPV3 has been implicated in EGFR signaling activation which is important for skin homeostasis^5^. To investigate whether TRPV3 activation-induced lytic cell death could result in the release of EGFR ligands like TGFα^5^, we quantified phosphorylated EGFR (pEGFR) in WT HeLa cells treated with conditioned medium (Supplementary Fig. S2a). Our results showed that pEGFR was detected in the cells treated with the medium from TRPV3-activated HeLa cells cultured in Ca^2+^ containing HBSS (Supplementary Fig. S2b). Furthermore, *Trpv3*^*G568V/+*^ mice, a mouse model of Olmsted syndrome with hyperkeratosis and sparse hair (Supplementary Fig. S2c, d)^6^, also exhibited increased levels of pEGFR downstream pERK in the epidermis (Supplementary Fig. S2e).

Lytic cell death is well established as a potent initiator of inflammation by releasing damage-associated molecular patterns (DAMPs) and pro-inflammatory cytokines^7^. In vitro, TRPV3-expressing mouse keratinocytes released TSLP^8^, a cytokine related to atopic dermatitis, in a carvacrol concentration-dependent manner (Supplementary Fig. S2f). We examined a panel of pro-inflammatory chemokines released from control and TRPV3-expressing mouse keratinocytes treated by carvacrol (Fig. 1h). The results showed that calcium-dependent lytic cell death was also accompanied by the release of multiple pro-inflammatory chemokines, which play important roles in inflammatory skin diseases (Fig. 1i; Supplementary Fig. S2g)^9–11^.

To investigate the functional consequences of increased pro-inflammatory cytokine release following TRPV3-mediated lytic cell death, we analyzed the immune response in carvacrol-treated skin in vivo. Upon TRPV3 activation with carvacrol, WT but not *TRPV3* KO mice exhibited dermatitis-like symptoms, with ear redness and swelling, increased ear thickness, and immune cell infiltration (Fig. 1j–l). We observed no significant increase in keratinocyte proliferation increase after carvacrol treatment (Supplementary Fig. S2h), but the expression of several chemokines, including *Ccl7/8* and *Cxcl1/2*, was up-regulated in keratinocytes isolated from ear skin treated with 3% carvacrol (Supplementary Fig. S2i–l). Additionally, *Trpv3*^*G568V/+*^ mice also exhibited increased immune cell infiltration (Supplementary Fig. S3). These findings collectively demonstrate that TRPV3 activation-induced lytic cell death leads to the release of inflammatory chemokines, disrupting skin immune homeostasis in vivo.

Treatment with inhibitors targeting known cell death mechanisms, including apoptosis, necroptosis, pyroptosis, and ferroptosis, failed to prevent calcium influx-induced lytic cell death (Supplementary Fig. S4a–j). Additionally, we examined the activation of critical executors associated with different types of cell death in the context of calcium influx-induced lytic cell death (Fig. 1m). In contrast to the positive controls, TRPV3-expressing HeLa cells treated with 2APB and camphor cocktail did not exhibit activation of caspase3 cleavage, cleaved N-GSDMD/E, RIPK1/RIPK3/MLKL phosphorylation, MLKL aggregation, or GPX4 decrease (Fig. 1n–r; Supplementary Fig. S4k). Furthermore, no cleaved caspase3 was observed in the skin of *Trpv3*^*G568V/+*^ mice skin in vivo (Supplementary Fig. S5a). We also employed genetic approaches to investigate the involvement of these pathways in *Trpv3*^*G568V/+*^ mice. Loss of *Caspase1/11* or *Mlkl* did not mitigate the skin phenotype observed in *Trpv3*^*G568V/+*^ mice, including sparse hair and immune cell infiltration (Supplementary Fig. S5b–j).

To identify the molecular executor responsible for calcium influx-induced lytic cell death, we performed a high-throughput chemical compound screen (Fig. 1s; Supplementary Fig. S6a, b). We screened approximately 3000 small-molecule compounds, including FDA-approved compounds and related molecules, in TRPV3-expressing mouse keratinocytes treated with 2APB and camphor cocktail. This screen’s top 3 validated hits were chlorhexidine hydrochloride, lercanidipine, and KN-93 phosphate (KN93). These compounds demonstrated dose-dependent rescue of calcium influx-induced lytic cell death (Fig. 1t–v; Supplementary Fig. S6c–g). Chlorhexidine and lercanidipine treatments inhibited calcium influx following TRPV3 activation, whereas KN93 did not (Fig. 1w). These results suggested that KN93 especially blocked a signal pathway downstream of calcium influx triggered by TRPV3 activation. Since KN93 is a CAMKII inhibitor, we found CAMKII inhibition by myristoylated autocamtide-2-related inhibitory peptide (Myr-AIP) also blocked calcium influx-induced lytic cell death (Supplementary Fig. S6h, i). These data suggested that TRPV3-mediated calcium influx induced lytic cell death through the CAMKII pathway. KN93 could not inhibit other types of cell death (Supplementary Fig. S6j–m), which distinguished calcium influx-induced lytic cell death from other death modalities.

We next investigated KN93’s potential to inhibit lytic cell death and subsequent immune response in vivo (Supplementary Fig. S6n). Topical application of KN93 significantly reduced keratinocyte membrane rupture in vivo (Fig. 1x). Additionally, pro-inflammatory chemokines, including CXCL1 and CXCL2, up-regulated by TRPV3 activation, exhibited significant reductions upon KN93 application in vivo (Fig. 1y, Supplementary Fig. S6o). Moreover, the ear thickness of mice with carvacrol-induced dermatitis was markedly reduced in a KN93 dosage-dependent manner (Fig. 1z).

In summary, we describe a novel calcium influx-induced lytic cell death mediated by TRPV3, distinct from known cell death mechanisms such as apoptosis, pyroptosis, ferroptosis, and necroptosis. We propose to name this novel form of lytic cell death as “calroptosis”, which is triggered by the activation of an ion channel and requires extracellular calcium influx/CAMKII pathway. In vivo, TRPV3-mediated calroptosis disrupts skin homeostasis and leads to immune cell infiltration, while KN93 can pharmacologically block TRPV3-mediated calroptosis and skin inflammatory response (Supplementary Fig. S7). Overall, our study provides clues about how keratinocytes sense and transmit signals to regulate tissue immune homeostasis and a potential treatment for skin inflammatory diseases.

### Supplementary information

Supplementary Information

## References

[1] Nicotera P, Bellomo G, Orrenius S (1992). Annu. Rev. Pharmacol. Toxicol..

[2] Smith GD (2002). Nature.

[3] Guo Y (2023). Biomolecules.

[4] Miyamoto T, Petrus MJ, Dubin AE, Patapoutian A (2011). Nat. Commun..

[5] Cheng X (2010). Cell.

[6] Song, Z. et al. *J. Investig. Dermatol*. 1–11 (2021).

[7] Anderton H, Wicks IP, Silke J (2020). Nat. Rev. Rheumatol..

[8] Wilson SR (2013). Cell.

[9] Saika A (2021). FASEB J..

[10] Islam SA (2011). Nat. Immunol..

[11] Brunner PM (2015). Exp. Dermatol..

