# Supplementary material for: Calcium influx-induced lytic cell death disrupts skin immune homeostasis
Source: Cell Discov. 2023 Dec 19;9:124. doi: 10.1038/s41421-023-00623-2 (PMC10728102; doi:10.1038/s41421-023-00623-2)
Supplement: Supplementary file 1 — Supplementary Information [file 41421_2023_623_MOESM1_ESM.pdf]

Figure S1

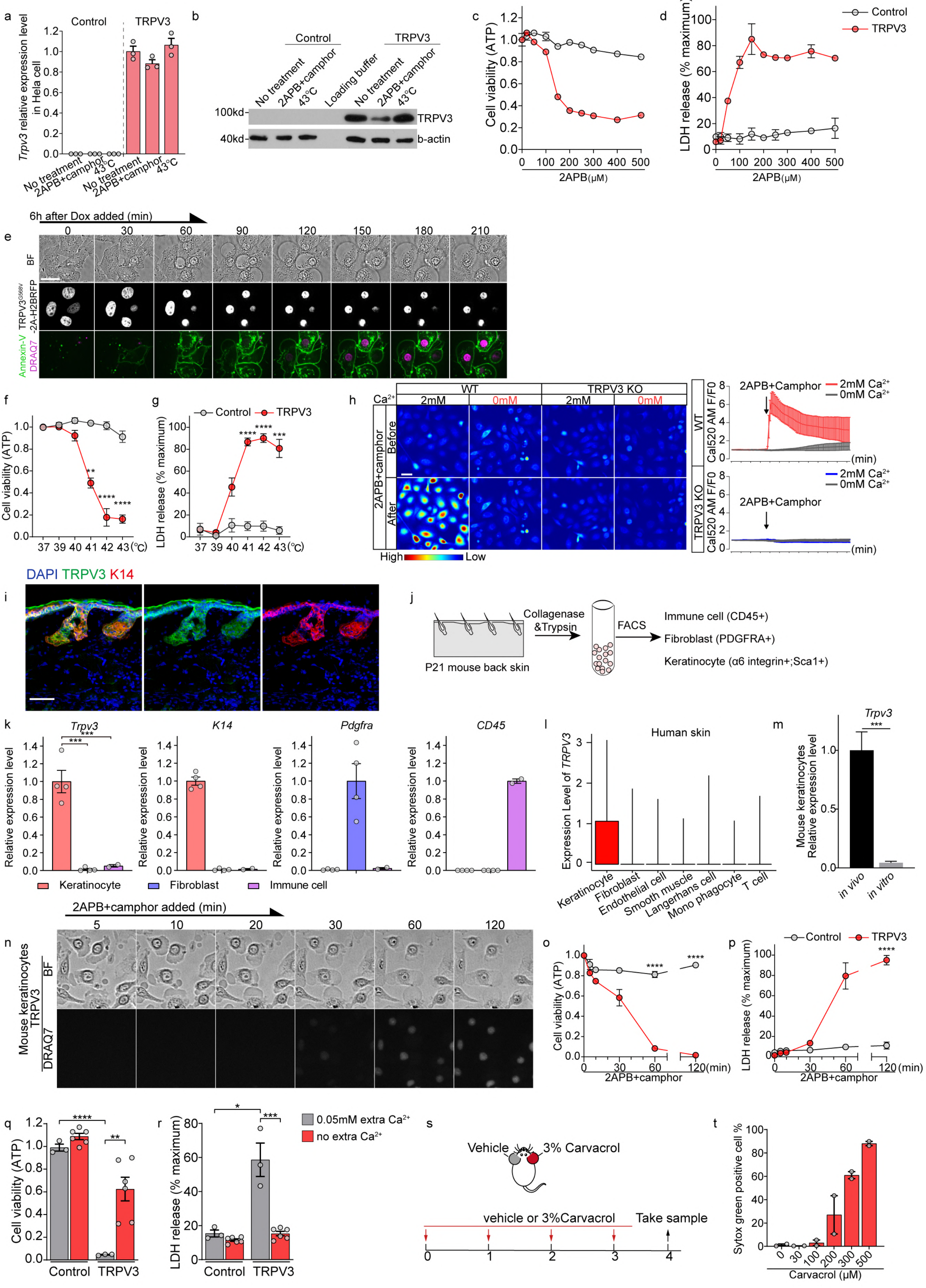

a
Control
TRPV3
Trpv3 relative expression level in Hela cell
No treatment
2APB+camphor
43°C
b
Control
TRPV3
No treatment
2APB+camphor
43°C
Loading buffer
100kd
TRPV3
40kd
b-actin
c
Cell viability (ATP)
2APB(μM)
d
LDH release (% maximum)
2APB(μM)
Control
TRPV3
e
6h after Dox added (min)
0
30
60
90
120
150
180
210
BF
TRPV3G568V-2A-H2BRFP
Annexin-V
DRAQ7
f
Cell viability (ATP)
(°C)
g
Control
TRPV3
LDH release (% maximum)
(°C)
h
Ca2+
WT
TRPV3 KO
2mM
0mM
2APB+camphor
Before
After
High
Low
2APB+Camphor
2mM Ca2+
0mM Ca2+
Cal520 AM F/F0
(min)
i
DAPI TRPV3 K14
j
P21 mouse back skin
Collagenase &Trypsin
FACS
Immune cell (CD45+)
Fibroblast (PDGFRA+)
Keratinocyte (α6 integrin+;Sca1+)
k
Trpv3
K14
Pdgfra
CD45
Relative expression level
Keratinocyte
Fibroblast
Immune cell
l
Human skin
Expression Level of TRPV3
Keratinocyte
Fibroblast
Endothelial cell
Smooth muscle
Langerhans cell
Mono phagocyte
T cell
m
Trpv3
Mouse keratinocytes Relative expression level
in vivo
in vitro
n
2APB+camphor added (min)
5
10
20
30
60
120
Mouse keratinocytes TRPV3
BF
DRAQ7
o
Cell viability (ATP)
2APB+camphor
(min)
p
Control
TRPV3
LDH release (% maximum)
2APB+camphor
(min)
q
Cell viability (ATP)
Control
TRPV3
r
LDH release (% maximum)
Control
TRPV3
0.05mM extra Ca2+
no extra Ca2+
s
Vehicle
3% Carvacrol
vehicle or 3%Carvacrol
Take sample
t
Sytox green positive cell %
Carvacrol (μM)

Figure S2

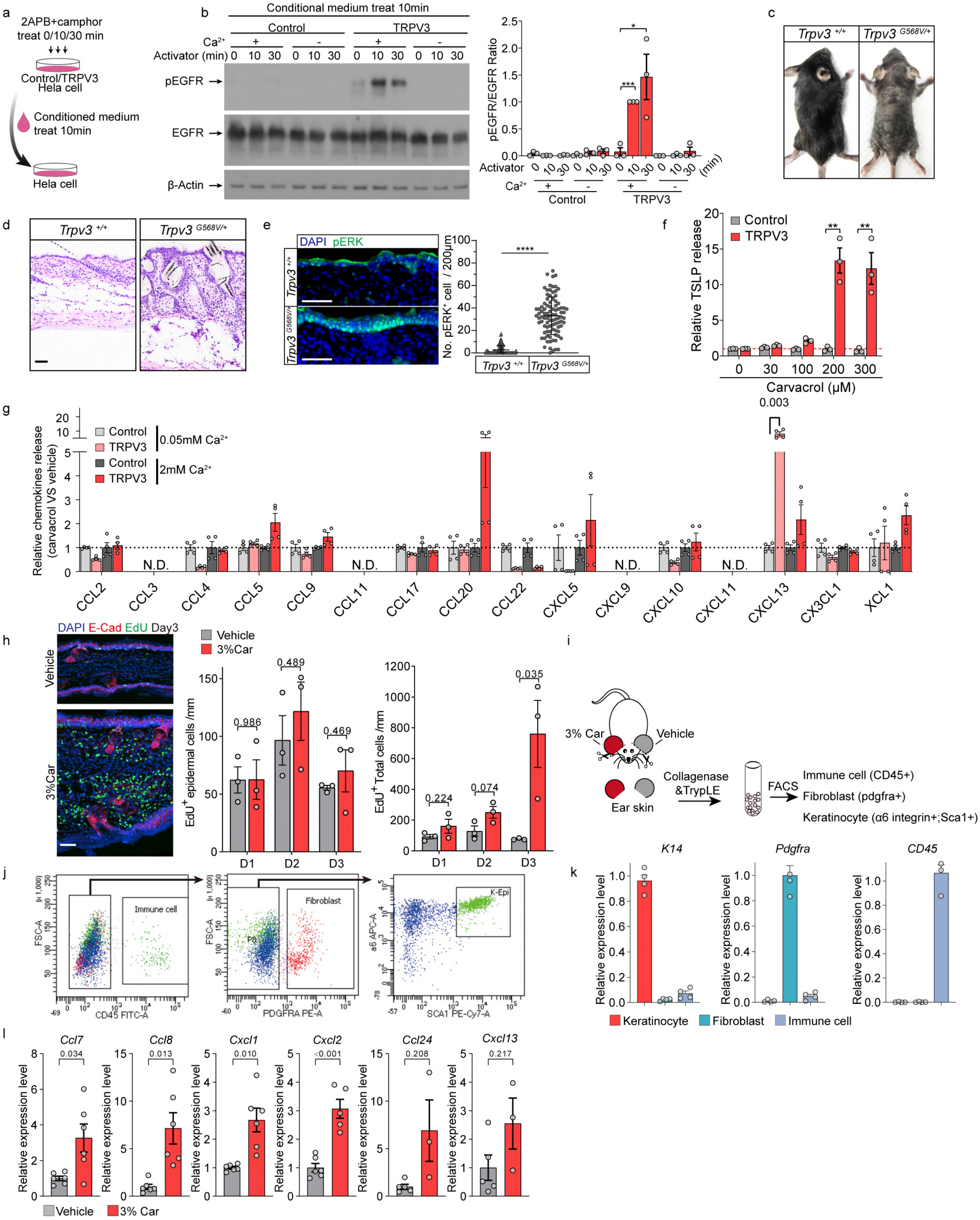
a
2APB+camphor treat 0/10/30 min
Control/TRPV3 Hela cell
Conditioned medium treat 10min
Hela cell
b
Conditional medium treat 10min
Control
TRPV3
pEGFR
EGFR
β-Actin
pEGFR/EGFR Ratio
Activator
c
d
e
DAPI pERK
No. pERK⁺ cell / 200μm
f
Control
TRPV3
Relative TSLP release
Carvacrol (μM)
g
Relative chemokines release (carvacrol VS vehicle)
0.05mM Ca²⁺
2mM Ca²⁺
N.D.
CCL2
CCL3
CCL4
CCL5
CCL9
CCL11
CCL17
CCL20
CCL22
CXCL5
CXCL9
CXCL10
CXCL11
CXCL13
CX3CL1
XCL1
h
DAPI E-Cad EdU Day3
Vehicle
3%Car
EdU⁺epidermal cells /mm
EdU⁺Total cells /mm
i
3% Car
Vehicle
Ear skin
Collagenase &TrypLE
FACS
Immune cell (CD45+)
Fibroblast (pdgfra+)
Keratinocyte (α6 integrin+;Sca1+)
j
Immune cell
Fibroblast
K-Epi
k
K14
Pdgfra
CD45
Relative expression level
Keratinocyte
Fibroblast
Immune cell
l
Ccl7
Ccl8
Cxcl1
Cxcl2
Ccl24
Cxcl13
Vehicle
3% Car

Figure S3

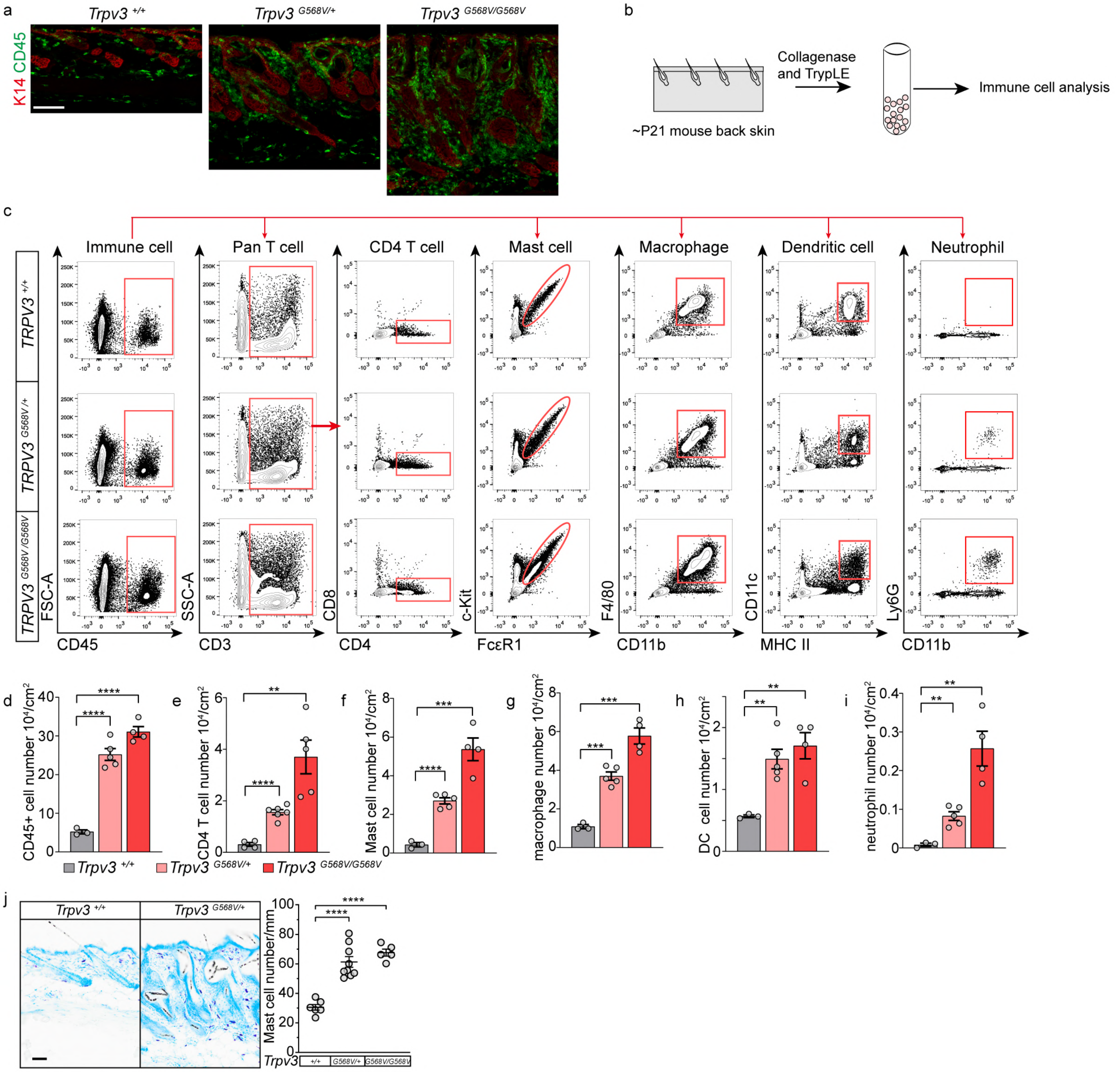

a
Trpv3 +/+
Trpv3 G568V/+
Trpv3 G568V/G568V
K14 CD45
b
Collagenase and TrypLE
~P21 mouse back skin
Immune cell analysis
c
Immune cell
Pan T cell
CD4 T cell
Mast cell
Macrophage
Dendritic cell
Neutrophil
TRPV3 +/+
TRPV3 G568V/+
TRPV3 G568V/G568V
FSC-A
CD45
SSC-A
CD3
CD8
CD4
c-Kit
FcεR1
F4/80
CD11b
CD11c
MHC II
Ly6G
CD11b
d
CD45+ cell number 10⁴/cm²
e
CD4 T cell number 10⁴/cm²
f
Mast cell number 10⁴/cm²
g
macrophage number 10⁴/cm²
h
DC cell number 10⁴/cm²
i
neutrophil number 10⁴/cm²
Trpv3 +/+
Trpv3 G568V/+
Trpv3 G568V/G568V
j
Trpv3 +/+
Trpv3 G568V/+
Mast cell number/mm
Trpv3
+/+
G568V/+
G568V/G568V

Figure S4

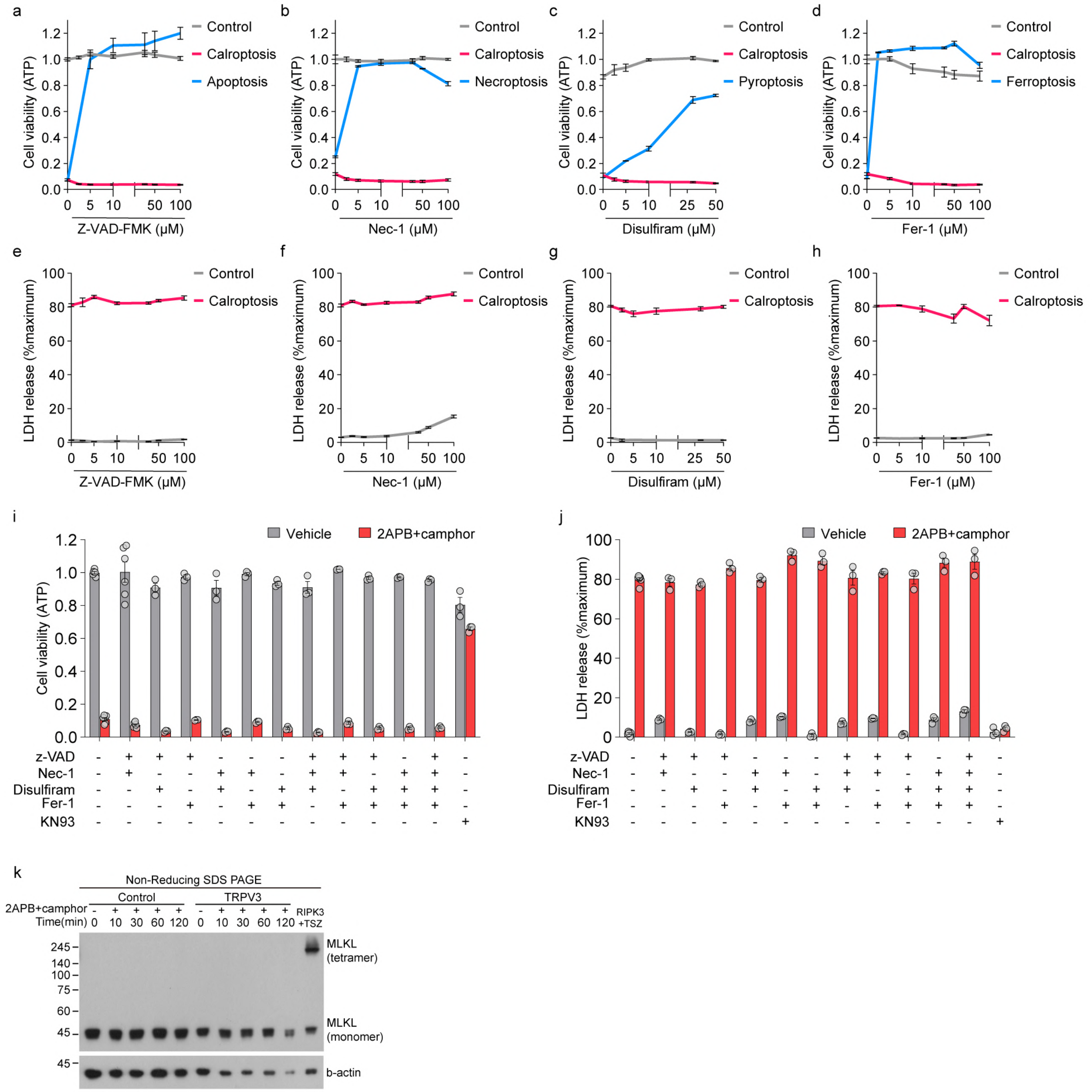
a
b
c
d
Cell viability (ATP)
Control
Calroptosis
Apoptosis
Necroptosis
Pyroptosis
Ferroptosis
Z-VAD-FMK (μM)
Nec-1 (μM)
Disulfiram (μM)
Fer-1 (μM)
e
f
g
h
LDH release (%maximum)
i
j
Vehicle
2APB+camphor
z-VAD
Nec-1
Disulfiram
Fer-1
KN93
k
Non-Reducing SDS PAGE
Control
TRPV3
2APB+camphor
Time(min)
RIPK3 +TSZ
MLKL (tetramer)
MLKL (monomer)
b-actin

Figure S5

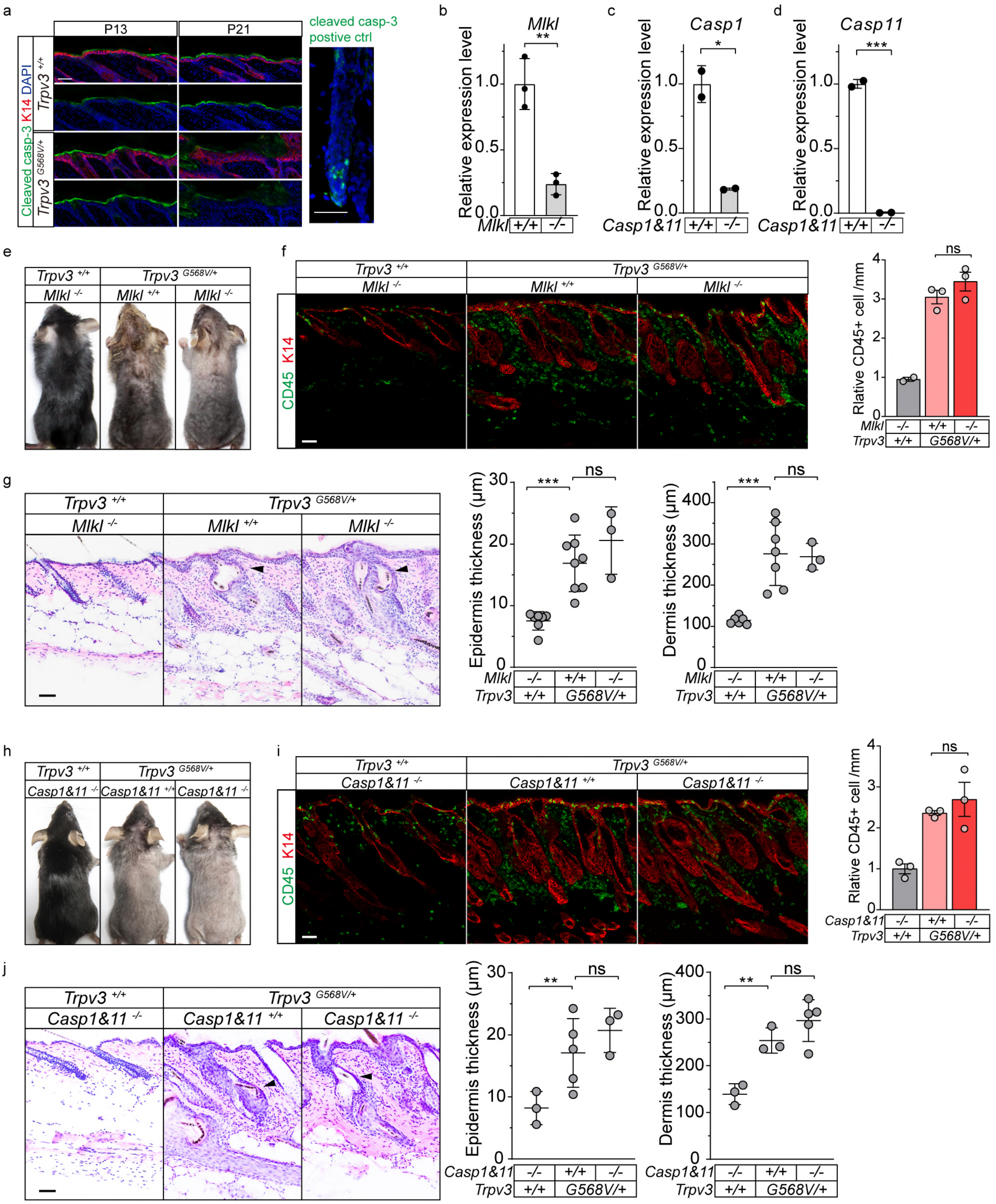

a
P13
P21
cleaved casp-3
postive ctrl
Cleaved casp-3 K14 DAPI
Trpv3 +/+
Trpv3 G568V/+
b
Mlkl
**
Relative expression level
1.0
0.5
0.0
Mlkl +/+ -/-
c
Casp1
*
Casp1&11 +/+ -/-
d
Casp11
***
e
Trpv3 +/+
Trpv3 G568V/+
Mlkl -/-
Mlkl +/+
Mlkl -/-
f
CD45 K14
ns
Rlative CD45+ cell /mm
Trpv3 +/+ G568V/+
g
***
ns
Epidermis thickness (μm)
Dermis thickness (μm)
h
Casp1&11 -/-
Casp1&11 +/+
i
j
**

Figure S6

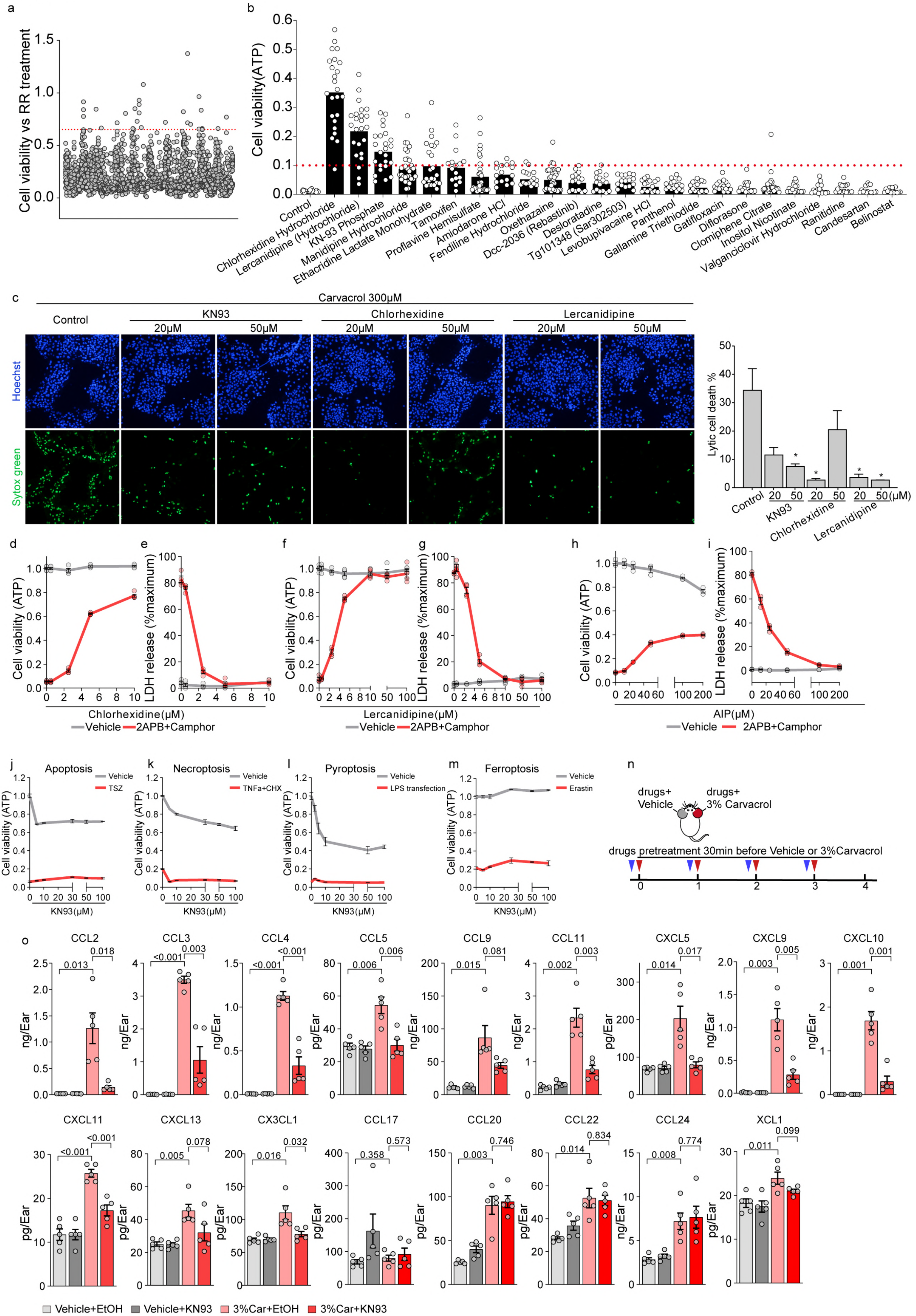

a
Cell viability vs RR treatment
b
Cell viability(ATP)
Control
Chlorhexidine Hydrochloride
Lercanidipine (Hydrochloride)
KN-93 Phosphate
Manidipine Hydrochloride
Ethacridine Lactate Monohydrate
Tamoxifen
Proflavine Hemisulfate
Amiodarone HCl
Fendiline Hydrochloride
Oxethazaine
Dcc-2036 (Rebastinib)
Desloratadine
Tg101348 (Sar302503)
Levobupivacaine HCl
Panthenol
Gallamine Triethiodide
Gatifloxacin
Diflorasone
Clomiphene Citrate
Inositol Nicotinate
Valganciclovir Hydrochloride
Ranitidine
Candesartan
Belinostat
c
Carvacrol 300μM
Control
KN93
Chlorhexidine
Lercanidipine
20μM
50μM
Hoechst
Sytox green
Lytic cell death %
(μM)
d
e
f
g
h
i
Cell viability (ATP)
LDH release (%maximum)
Chlorhexidine(μM)
Lercanidipine(μM)
AIP(μM)
Vehicle
2APB+Camphor
j
Apoptosis
TSZ
k
Necroptosis
TNFa+CHX
l
Pyroptosis
LPS transfection
m
Ferroptosis
Erastin
KN93(μM)
n
drugs+ Vehicle
drugs+ 3% Carvacrol
drugs pretreatment 30min before Vehicle or 3%Carvacrol
o
CCL2
CCL3
CCL4
CCL5
CCL9
CCL11
CXCL5
CXCL9
CXCL10
CXCL11
CXCL13
CX3CL1
CCL17
CCL20
CCL22
CCL24
XCL1
ng/Ear
pg/Ear
Vehicle+EtOH
Vehicle+KN93
3%Car+EtOH
3%Car+KN93

Figure S7

a

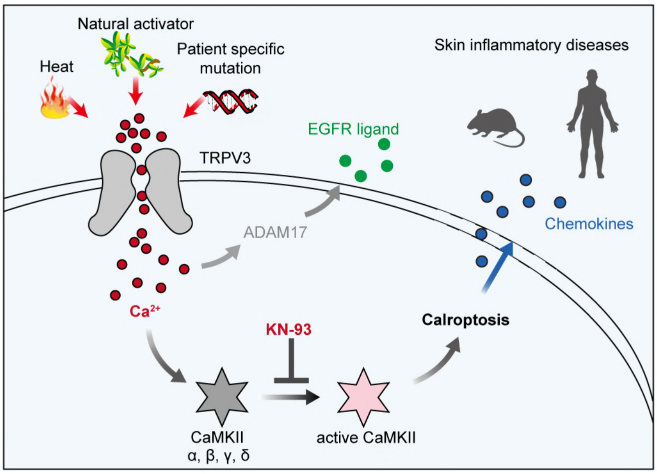
Natural activator
Heat
Patient specific mutation
Skin inflammatory diseases
TRPV3
EGFR ligand
ADAM17
Chemokines
Ca2+
KN-93
Calroptosis
CaMKII
α, β, γ, δ
active CaMKII

Supplementary figure legends

Supplementary Fig. S1

(a) qPCR of TRPV3 of HeLa cells. The TRPV3 primer could distinguish both mouse and human TRPV3. **(b)** Western blot of TRPV3 of HeLa cells. After 2APB treated HeLa cells (Control, gray line) or mTRPV3 expressed HeLa cells (TRPV3, red line) for 2h, cell viability**(c)** was assessed by CellTiter-Glo luminescent assay (n=3), and membrane integrity**(d)** was measured by LDH release (n=3). **(e)** Live images of HeLa cells expressed dox-inducible mouse TRPV3 G568V mutant-2A-H2BRFP after dox induction for the indicated time. H2BRFP (white) indicated TRPV3 G568V mutant expression. Membrane blebbing and nuclear condensation were shown in bright fields (BF). Annexin V (green) indicated phosphatidylserine externalization, and DRAQ7 (purple) presented cell membrane integrity disruption. Scale bar:20μm. Cell viability (n=4) **(f)** and membrane integrity (n=4) **(g)** of HeLa cells (Control, gray line) or mTRPV3 expressed HeLa cells (TRPV3, red line) cultured in the temperature range from 37°C to 43°C for 3 hours. **(h)** Calcium imaging of WT or TRPV3 KO mouse primary keratinocytes with 2mM or 0mM extracellular calcium before and after 2APB and camphor cocktail treatment. F/F0 of single cells in each group (n>40) was shown. Scale bar:20μm. **(i)**TRPV3 and K14 staining of mouse ear skin section. Scale bar: 50μm. **(j)** Workflow of isolation of different types of cells from mouse skin by FACS. **(k)** qPCR of isolated skin cells by FACS. **(l)** Expression level of TRPV3 analysis in healthy human skin single-cell seq data. The lower and upper hinges indicated 1% and 99% percentile value. **(m)** TRPV3 qPCR of mouse keratinocytes cultured *in vitro* and obtained *in vivo*. **(n)**Live images of mouse keratinocytes expressed mouse TRPV3

treated with 2APB and camphor cocktail during the indicated time. DRAQ7 (white) presented cell membrane integrity disruption. Scale bar:20μm. Cell viability**(o)** (n=3) and membrane integrity**(p)** (n=3) of mouse keratinocytes treated by 2APB and camphor. Cell viability (n>=3) **(q)** and membrane integrity **(r)** (n>=3) of wild-type mouse keratinocytes cells (Control) or mTRPV3 expressing keratinocytes (TRPV3-Keratinocytes) after 2APB and camphor cocktail treatment with or without extracellular calcium. **(s)** Schematic of carvacrol-induced TRPV3 activation *in vivo*. **(t)**Sytox green indicated lytic cell death of mTRPV3 expressing keratinocytes treated with carvacrol for 6 hours(n=2).

Supplementary Fig. S2

(a) Conditioned medium from HeLa cell treated by 2APB and camphor incubate to HeLa cell. **(b)** Western blot analysis of HeLa cells treated by the conditioned medium. Quantification of the relative protein level (n = 3). **(c)** Representative pictures of *Trpv3*$^{+/+}$ and *Trpv3*$^{G568V/+}$ P21 mice. **(d)** Hematoxylin and eosin analysis of back skin sections from *Trpv3*$^{+/+}$ and *Trpv3*$^{G568V/+}$ P21 mice. Scale bar: 50μm. **(e)** pERK staining(green) of *Trpv3*$^{+/+}$ *or Trpv3*$^{G568V/+}$ P21 mice skin. Scale bar: 50μm. Quantification of pERK-positive cell number per 200μm (n = 3). **(f)** ELISA of TSLP release from mouse keratinocytes treated with carvacrol for 6 hours(n=3). **(g)** Pro-inflammatory chemokines relative release of mouse keratinocytes treated with 300μM carvacrol for 6 hours using vehicle-treated as control. Cells were cultured in 0.05mM or 2mM extracellular calcium concentration. **(h)** EdU labeling of wild-type mouse ear skin treated by vehicle or carvacrol at day 1 to day3. Scale bar: 50μm **(i-j)** Workflow of isolation of different types of cells from mouse ear skin by FACS. **(k-l)** qPCR of isolated

mouse ear skin cells by FACS.

Supplementary Fig. S3

(a) Immunofluorescent staining of K14(red) and CD45(green) of *Trpv3*$^{+/+}$, *Trpv3*$^{G568V/+}$ and *Trpv3*$^{G568V/G568V}$ P21 back skin section. Scale bar: 100μm. **(b)** Mouse back skins were digested by collagenase D and TrypLE for flow cytometry. **(c)** Gating strategies for immune cells of mice back skins. **(d-i)** Immune cell numbers per cm^2 of *Trpv3*$^{+/+}$, *Trpv3*$^{G568V/+}$*, and Trpv3*$^{G568V/G568V}$ P21 back skin measured by flow cytometry (n>=3). **(j)** Toluidine Blue staining of *Trpv3*$^{+/+}$*, Trpv3*$^{G568V/+}$*,* and *Trpv3*$^{G568V/G568V}$ P21 back skin section. Scale bar: 50μm. Quantification of mast cell number per 1mm. (n >=5). Data are presented as mean ± SEM.

Supplementary Fig. S4

Cell viability (n>=3) **(a-d)** and membrane integrity (n>=3) **(e-h)** were measured in mouse keratinocytes expressed mTRPV3 when treated by vehicle or 2APB+camphor (Calroptosis) for 2 hours with the indicated concentration of compounds treatment and 1 hour pre-treatment. TNFa and CHX (cycloheximide) treated HeLa cells acted as apoptosis positive control. HeLa expressed RIPK3 stimulated by TNFα (T), Smac mimetic (S), and a pan-caspase inhibitor zVAD (Z) (TSZ) acted as necroptosis positive control. Lipopolysaccharide (LPS) electroporation of HeLa cells served as pyroptosis positive control. Erastin treated 3T3 cells acted as ferroptosis positive control. Cell viability (n>=3) **(i)** and membrane integrity (n>=3) **(j)** were measured in mouse keratinocytes expressed mTRPV3 when treated by vehicle or 2APB+camphor with 50μM of each death inhibitors or KN93. **(k)** MLKL aggregation analysis of control or mTRPV3 expressing HeLa cell treated by 2APB and camphor with indicated time.

Supplementary Fig. S5

(A) Immunofluorescent staining of K14(red) and cleaved caspase3 (green) of *Trpv3*$^{+/+}$ and *Trpv3*$^{G568V/+}$ back skin section. Scale bar: 50μm. **(b-d)** *Mlkl, Caspase 1*, and *Caspase 11* mRNA levels of the skin tissue from indicated strains were measured by qPCR. **(e, h)** Representative pictures of the indicated strains. **(g, j)** Hematoxylin and eosin analysis of back skin sections from the indicated strains. Black arrows indicated abnormal hair follicles. Scale bar: 50μm. Epidermis and dermis thickness was measured (n >=3). **(f, i)** Immunofluorescent staining of K14(red) and CD45(green) of indicated strains back skin section and statistics of relative CD45 positive total immune cell number per micrometer of the sections(n=3). Scale bar: 50μm.

Supplementary Fig. S6

(a) Cell viability after each compound treatment compared to ruthenium red (RR) in the primary screen of 3000 compounds. **(b)** The top 30 hits from the primary screen were re-screened following the chemical compound screen workflow. **(c)** Representative images of mouse keratinocytes expressed mTRPV3 treated with 300μM carvacrol with the indicated concentration of compounds. Total cell nuclei were stained by Hoechst (blue). Lytic cells were indicated by sytox green(green). The percentages of sytox green positive cells indicated as lytic death cells in total cells were recorded of mouse keratinocytes expressed mTRPV3 treated with 300μM carvacrol with the indicated concentration of compounds. **(d-i)** Cell viability (n>=3) and membrane integrity (n>=3) were measured in mouse keratinocytes expressed mTRPV3 when treated by vehicle or 2APB+camphor with the indicated concentration of compounds. **(j-m)** Cell viability (n>=3) were measured when cells were induced to

different types of regulated cell death and co-treated with the indicated concentration of KN93. TNFa and CHX (cycloheximide) treated HeLa cells for 10 hours acted as apoptosis positive control. HeLa expressed RIPK3 stimulated by TNFα (T), Smac mimetic (S), and a pan-caspase inhibitor zVAD (Z) (TSZ) for 10 hours acted as necroptosis positive control. HeLa cells treated by Lipopolysaccharide (LPS) electroporation served as pyroptosis positive control. Erastin treated 3T3 cells for 12 hours acted as ferroptosis positive control. **(n)** Schematic diagram of carvacrol-induced TRPV3 activation in mouse ear treated with KN93. **(o)** The protein levels of pro-inflammatory chemokines in mouse ear after indicated treatments on day 3.

Supplementary Fig. S7

(a) The summary diagram of calroptosis. Activation of TRPV3 by different stimuli triggers a novel calcium influx-induced lytic cell death "calroptosis" and contributes to skin inflammation through pro-inflammatory chemokines release. Pharmacological inhibition with KN93 can block this process through CaMKII. The release of EGFR ligand also increases in this condition, which is important in skin homeostasis.

Methods

Mice

C57BL/6N mice were purchased from Charles River. Olmsted syndrome like Trpv3 knock-in mice (*Trpv3* $^{+/G568V}$) and TRPV3 KO mice were kindly provided by Dr. Yong Yang[1,2]. *Mlkl-/-* mice were kindly provided by Dr. Xiaodong Wang. *Casp1/11-/-* mice were kindly provided by Dr. Feng Shao. All mice were bred and maintained in a specific pathogen-free facility in accordance with the Guide for the Care and Use of Laboratory Animals of the National Institutes of Biological Sciences (NIBS).

Cell Culture

HeLa cells and NIH/3T3 were obtained from the American Type Culture Collection (ATCC) and cultured in DMEM medium supplemented with 10% (v/v) FBS and 1% (v/v) Penicillin-Streptomycin. 293FT cells (Thermo Fisher Scientific, Cat #R70007) used for the lentivirus package were maintained in DMEM medium supplemented with 10% (v/v) FBS, 1% (v/v) Penicillin-Streptomycin, 1% (v/v) L-glutamine, 1% (v/v) 100mM sodium pyruvate, 1% (v/v) 7.5% sodium bicarbonate, and 500 mg/mL G418 (Gibco). Lentivirus was packaged as previously described[3]. Mouse keratinocytes were isolated and established as previously reported[4]. All cell lines were cultured at 37°C in a cell incubator with 5% CO2 except for cells under heat-activation assay.

For generation of mouse TRPV3 overexpressing HeLa cells, wild-type HeLa cells were infected with lentivirus-TetOn-mouse TRPV3-2A-H2BRFP or lentivirus-TetOn-H2BRFP to generate TRPV3 HeLa or control HeLa respectively. Expression of mouse TRPV3 were induced before all experiments by 4μg/ml Doxycycline for more than 24 hours.

For generation of mouse TRPV3 G568V overexpressing HeLa cells, wild-type HeLa cells were infected with lentivirus-TetOn-mouse $TRPV3^{G568V}$-2A-H2BRFP to generate $TRPV3^{G568V}$ HeLa.

For generation of mouse TRPV3 overexpressing mouse keratinocytes, TRPV3 KO mouse keratinocytes were infected with lentivirus-mouse TRPV3-2A-H2BRFP or lentivirus-H2BRFP to generate TRPV3 mouse keratinocytes or control keratinocytes respectively.

The following drugs for cell death induction and inhibition were used: TNFa

(Biolegend), CHX (kindly provided by Dr. Xiaodong Wang.), Smac mimetic (kindly provided by Dr. Xiaodong Wang.), Z-VAD-FMK, Fer-1, Nec-1 (GlpBio), LPS (Sigma), Erastin, Disulfiram (Selleck).

Heat-activation assay

Custom-designed temperature control plates were used in heat-activation assay. The temperature control plate consisted of a thermos-electric cooler (TEC) to offer controllable temperature on one side which fit into the bottom of cell culture plate, using water circulation on the other side as the heat sink. The real-time temperature of the culture medium was detected by a Pt100 temperature sensor and return to the programmable logic controller which control the TEC to achieve closed loop temperature control. The cell culture temperature accuracy is ±0.2°C. The temperature control plates were set in the cell incubator with 5% CO2. We used the heat plates to treat TRPV3 or control HeLa cells in different temperatures vary from 37°C to 43°C for 3 hours.

Live imaging of cell death morphology

HeLa cell or mouse keratinocytes were plated in 35mm cell culture dish with glass bottom (NEST). Before imagining, cell medium was replaced by fresh medium with DRAQ7(1.5μM, Abcam) and Annexin V-FITC (1:500 dilution, Invitrogen). For imaging cells treated by 2APB and camphor, after pretreatment of DRAQ7 and Annexin V-FITC for 15min, 2APB and camphor were added and videos were immediately recorded for more than 2 hours. For imaging cells with dox-induced TRPV3 G568V expression, 4ug/ml Dox was added 6h before imaging. Cell medium was replaced by fresh medium with DRAQ7, Annexin V-FITC and dox 15min before video recording. Cell live imaging

videos were taken using a PerkinElmer Ultra VIEW spinning disk microscope and processed by image J. All image data are representative from at least three randomly selected fields.

Measuring cell viability and membrane leakage

Cell viability was measured with the CellTiter-Glo luminescent viability assay (Promega) according to the manufacturer's instructions. Membrane leakage of cells was measured by LDH releasing using the CytoTox96® non-radioactive cytotoxicity assay (Promega) according to the manufacturer's instruction. Briefly, HeLa cells were plated in 48-well culture plates at 10000 cells per well with 4ug/ml dox 48hours before the assay. Mouse keratinocytes were plated in 48-well culture plates at $2.5x10^4$cells per well 20 hours before the assay. After activator or heat treatment, 50μl culture medium were pipetted in to a new 96well white microplate with clear flat bottom and mixed with assay buffer 50μl CytoTox 96® reagent, then incubate the mixture 15-30min at room temperature. Finally, add 50μl stop solution to each well and record the absorbance at 492nm. CellTiter-Glo assay reagent was added into cells with the rest of culture medium and incubated 15min at room temperature. Then, the luminescent signals were immediately recorded. The absorbance and luminescent signal were recorded in a plate reader (PerkinElmer, EnSpire).

Membrane leakage was also measured by sytox green staining. Cells were incubated with medium containing Sytox green (1μM, Invitrogen) and Hoechst 33342(1μg/ml, Invitrogen) for 30min and imaged by Olympus IX51 inverted fluorescence and phase contrast tissue culture microscope. The percentage of sytox green positive cell in total Hoechst positive cell was calculated as the percentage of lytic cell death.

In vitro Ca2+ imaging

For calcium imaging of 2APB and camphor cocktail treated mouse WT and TRPV3 KO primary keratinocytes with or without extracellular calcium. Mouse WT and TRPV3 KO primary keratinocytes were isolated from P1-P3 back skin as previous described[4]. Cells were incubated with 2 mM Cal520-AM (AAT Bioquest) in culture medium at 37°C for 60 min. Then, cells were washed with no Ca^{2+} HBSS, then cultured in HBSS with or without 2mM calcium. Fluorescence at a wavelength of 488 was recorded using a spinning disk microscope at 1 frame per 5 s. First fluorescence was recorded for 3 min. Then after 2APB and camphor were added into the culture medium, fluorescence was recorded for another 10 min.

For calcium imaging of mouse keratinocytes following TRPV3 activation with drugs co-incubation. Mouse TRPV3 expressing keratinocytes were incubated with 2 mM Cal520-AM in culture medium at 37°C for 30 min and drugs (10μM chlorhexidine, 50μM lercanidipine and 100μM KN93) with cal520-AM for another 30min. Then, cells were washed with no Ca^{2+} HBSS, then cultured in HBSS with drugs of indicated concentration. Fluorescence at a wavelength of 488 was recorded using a spinning disk microscope at 1 frame per 5 s. First fluorescence was recorded for 3 min. Then drugs plus 2APB and camphor were added into the culture medium, fluorescence was recorded for another 10 min.

The fluorescence intensity was calculated and processed in ImageJ.

Carvacrol-induced dermatitis mouse model

For activation TRPV3 *in vivo*, 8-10 weeks female C57BL/6N mice or TRPV3 KO mice were used for carvacrol-induced dermatitis mouse model[5]. 3% carvacrol (sigma) was

dissolved in 50%EtOH and topically applied 25μl twice a day with a 15-30 minutes interval on both side of mouse right ear， while vehicle were applied on the left ear. Carvacrol was treated for four consecutive days and ear thickness was recorded by a vernier caliper every day. Ear samples were taken for further analysis after 3 or 4days treatment. For KN93 (Selleck) pretreatment, KN93 phosphate was dissolved in 50%EtOH and topically applied 20μl once a day 30min before first carvacrol treatment on both side of mouse ear. 25mg/kg/ear KN93 was used in all experiments unless there is an additional note.

***In vivo* lytic cell death labelling**

1% Evens blue dye[6] was dissolved in PBS and injected in to mice 5μl/g by retro-orbital injections 24 hours before sample taken. Fresh skin samples were immediately embedded in O.C.T. compound (Tissue-Tek), frozen on dry ice, and cryo-sectioned (20μm). Sections were fixed for 10 min in 4% (v/v) paraformaldehyde in PBS, and blocked for 1 hour in a non-permeabilized blocking buffer (2% normal donkey serum and 1% BSA in PBS). The antibodies were dissolved in non-permeabilized blocking buffer too. The primary antibody (anti-E Cadherin antibody, Abcam, ab11512) was incubated 1hour at room temperature, followed by washing three times with PBS for 10min. The secondary antibody was incubated with the samples at room temperature for 1 hour, followed by three washes with PBS for 15 min each. Then the slides were mounted and prepared for imagining. The slides were imaged on a Nikon A1 confocal microscope. Microscopy data were analyzed using Bitplane Imaris and Image J.

***In vivo* EdU labelling**

Carvacrol-induced dermatitis mouse model were used described above. 24h before

take sample for EdU detection， Mice was intraperitoneal injected 50mg/kg EdU (MCE # HY-118411) in PBS. After 24h, fresh skin was embedded in O.C.T. compound, frozen on dry ice, and cryo-sectioned (20-25μm). EdU detection of the skin section was based on click reaction using EdU image kit (Abbkine #KTA2030). Immunofluorescence staining was performed after EdU-Click assay. sections were imaged on a Nikon A1 confocal microscope. Microscopy data were analyzed using Bitplane Imaris.

Immunofluorescence staining

For section staining, skin tissues were embedded in O.C.T. compound, frozen on dry ice, and cryo-sectioned (20-25μm). Sections were fixed for 10 min in 4% (v/v) paraformaldehyde in PBS at room temperature or in acetone at -20°C and blocked for 1 h in blocking buffer (2% normal donkey serum, 1% BSA, and 0.5% Triton in PBS). The antibodies were dissolved in the blocking buffer too. The primary antibodies were incubated overnight at 4°C, followed by washing three times with PBS for 15 min. The secondary antibodies were incubated with the samples at room temperature for 1 hours, followed by three washes with PBS for 15 min each. Immunofluorescence staining slides were imaged on a Nikon A1 confocal microscope. Microscopy data were analyzed using Bitplane Imaris and Image J. The following antibodies were used: anti-TRPV3(Alomone, ACC-033), anti-pERK(CST #9101), anti-K14(lab made).

Hematoxylin and eosin staining

For Hematoxylin and eosin (H&E) staining, skin samples were cyto-sectioned (15μm) and fixed in 4% paraformaldehyde in PBS for 10 min, followed by three washes with PBS for 10min. Then, sections were stained with Hematoxylin (Sigma) for 10-20 s and then rinsed in water and 0.3% acid alcohol for clear signal, followed by staining with

Eosin (Sigma) for 30 s. Sections were next dehydrated in alcohol and cleared in xylene. The slides were mounted by neutral balsam (Solarbio). H&E staining images were collected using a VS120 microscope. Microscopy data were analyzed using Image J.

Toluidine Blue staining

For mast cell staining, we used toluidine blue. Samples were sectioned and fixed same as H&E staining. Then, stain sections in toluidine blue working solution for 2-3 minutes and wash in waters for three times. Sections were next dehydrated in alcohol and cleared in xylene. The slides were mounted by neutral balsam. Toluidine blue should stain the mast cells purple and the background blue. Toluidine Blue staining images were collected using a VS120 microscope.

Western blot

Cell lysates were prepared using SDS-PAGE sample loading buffer (Beyotime, P0015L) or Non-reducing SDS loading buffer. Proteins were separated by SDS-PAGE and transferred onto a PVDF membrane (Millipore). Western blot analysis of target proteins was conducted using the corresponding primary antibodies diluted with TBST containing 5% skim milk, followed by exposure to an HRP-conjugated secondary antibody. The reactive bands were visualized using ECL Plus reagents (Clinx), and the relative intensity of reactive bands was analyzed using ImageJ. The following antibodies were used: anti-EGFR (Abcam, ab52894), anti-phos-EGFR (Cell signaling, 2234s), anti-MLKL (Abcam, ab184718), anti-phos-MLKL (Abcam, ab196436), anti-RIPK1(Cell signaling, 3493), anti-phos-RIPK1(Cell signaling, 65746), anti-RIPK3(Cell signaling, 13526), anti-phos-RIPK3(Cell signaling, 93654), anti-b-Actin (MBL, pm053-7), anti-GSDME (Abcam, ab215191), anti-full length-GSDMD (Abcam, ab210070),

anti-N terminal-GSDMD (Abcam, ab215203), anti-GPX4(Abcam, ab125066), anti-TRPV3(Abcam, 85022).

TSLP ELISA

Mouse keratinocytes were cultured in E medium with 2mM calcium 40 hours before treatment and then medium were refresh by E medium with 2mM calcium and indicated concentrations of carvacrol(sigma). 6 hours later, cell death was indicated by sytox green and cell supernatants were collected and filtered through a 0.22μm PVDF membrane (Millipore, SLGPR33RB) to remove cell debris. Mouse TSLP ELISA kits were purchased from Biolegend (catalog 434107) and TSLP in cell supernatant was detected according to the manufacturer’s instructions. The standard curve was included for each experiment.

Multiple chemokines detection

For cell supernatant detections，mouse keratinocytes were cultured in E medium with 2mM calcium or 0.05mM calcium 40 hours before treatment and then medium were refresh by E medium with 2mM calcium or 0.05mM calcium and 300μM carvacrol(sigma). 6 hours later, cell supernatants were collected and filtered through a 0.22μm PVDF membrane to remove cell debris. Supernatant were concentrated to 20X using Microsep advance centrifugal devices with 3K omega membrane (Pall, MCP003C41) following the manufacturer’s instruction. Supernatant with or without concentration were used to detect multiple pro-inflammatory chemokines through Mouse pro-inflammatory chemokine panel-13plex (Biolegend, 740451) and Mouse pro-inflammatory chemokine panel 2-8plex (Biolegend, 741068) according to the manufacturer’s instructions. In brief, supernatant was incubated with a capture bead

mixture. Each bead set is conjugated with a specific chemokine antibody on the surface and serves as the capture bead for a particular chemokine, and the different bead set could be clearly distinguished from each other by different sizes and levels of APC fluorescence. After incubation, the beads were washed to remove unspecific binding. Then, biotinylated detection antibodies were added, which would bind to its specific analyte bound on the capture beads to form capture bead-analyte-detection antibody sandwiches. Finally, streptavidin-phycoerythrin (SA-PE) was added, which would bind to the biotinylated detection antibodies, providing fluorescent signal with intensities in accordance with the amount of bound analyte. The standard and sample corresponding beads were recorded by the BD FACSAria™ fusion flow cytometer. Each chemokine concentration in supernatant was analyzed using the LEGENplex™ data analysis software. The standard curve was included for each experiment.

For mouse skin tissue protein detection, the fresh skin was frozen into liquid nitrogen, and ground into powder using a low temperature grinding instrument (Shanghai Jingxin). The protein was extracted using RIPA lysis buffer (Invitrogen #89901). The chemokines were detected using mouse pro-inflammatory chemokine panel-13plex (Biolegend, 740451) and mouse pro-inflammatory chemokine panel 2-8plex (Biolegend, 741068) according to the manufacturer's instructions.

Flow cytometry analysis of skin immune cell

Mouse back and ear skin immune cells were isolated using collagenase D(Roche) and TrypLE (Gibco) or Trypsin (Gibco). For mouse back skin immune cell analysis, $1cm^2$ mouse back skin was cut and floated, dermis side down, on 3mg/ml collagenase D with 100μg/ml DNase I(Roche) dissolved in HBSS (sigma) at 37°C, 80rpm, for 1-2

hours. Dermis fractions were scraped gently, and the total cell mixtures were transferred to a new 50ml centrifuge tube and replenished up to 50ml using 5%FBS in PBS. Then, the digested tissues were centrifuged and resuspended in TrpLE(Gibco) or Trypsin (Gibco) and incubated at 37°C, 80rpm, 20 min. Single-cell suspensions were obtained by pipetting gently and filtering through 70 mm strainers and 40 mm strainers. 5% FBS was added to inactivate TrpLE, and cells were collected by centrifugation for 5 min at 350g. Cell suspensions were incubated with antibodies, 20 min at 4°C with gentle shaking. For mouse ear skin immune cell analysis, ear skin was split into two halves and taken off cartilage using tweezers. The following steps were same as back skin treatment.

The following antibodies were used: Anti-CD45 (Biolegend, 103108), Anti-CD3 (Biolegend, 100220), Anti-CD4 (Biolegend, 100408), Anti-CD8 (Invitrogen, 17008183), Anti-c-kit (PE, 553355), Anti-FceR1a (Biolegend, 134316), Anti-CD11b (Biolegend, 101216), Anti-F4/80 (Biolengend, 123116), Anti-MHCII (Biolegend, 107630),Anti-CD11C (Biolegend, 117310),Anti-Ly6G (Biolegend, 127624), anti-pdgfra (Biolegend #135906), anti-CD49f(ebioscience #17-0495-82), anti-Sca1(Biolegend #108114). Antibodies described above were used with the dilution rate 1:300 incubated on ice for 15-30 min.

Cell analysis were performed on BD FACSAria^{TM} flow cytometer equipped with FACSDiva software (BD bioscience). FACS analyses were performed using FlowJo software (FlowJo LLC).

RNA extraction and Real-time PCR

For skin tissue RNA extraction and real-time PCR, 0.5cm^2 back skin was frozen in to

liquid nitrogen, and ground into powder using a low temperature grinding instrument (Shanghai Jingxin). The tissue powder was added with Trizol (Life Technologies) followed by extraction using a Direct-zol RNA Miniprep Kit (Zymo research).

For small numbers of cells obtained from FACS, RNA extraction was conducted using single cell RNA Purification Kit (Norgen Biotek #ngb-51800) according to the manufacturer's instructions.

To obtain cDNA, equal amounts of RNA were added to reverse-transcriptase reaction mix (Vazyme, R222-01). Real-time PCR was conducted using a CFX96TM Real-Time system (Bio-Rad) with SYBR FAST qPCR Kit (KAPA).

Chemical compound screen workflow and validation

Mouse keratinocytes were plated 4000 per well into a 384 plate (Corning, 3765) with 50μl E medium. After 20 hours cultured at 37°C in a cell incubator with 5% CO2, using 0.5μl 1mM drugs were added to each well with final concentration is 10μM. Pretreatment for 4 hours then add 10μl 500μM 2APB and 10mM camphor cocktail. 2 hours later, 15μl CellTiter-Glo luminescent viability assay buffer was added to each well and luminescent signals were recorded immediately in a plate reader (PerkinElmer, EnSpire). A ~3000 drug related compound library used for the screen was obtained from Chemistry center of NIBS. There are two DMSO only (negative control) columns and two ruthenium red (RR, positive Control) columns for each plate to normalize the inter-plates variation. We first pick up primary thirty hits, which cell viabilities reached 60% of the positive control group. The primary hits were validated following same steps of compound screen workflow. For top 3 hits validation with different concentrations, 2.5x 10^4 mouse keratinocytes were plated into each well of

48 well plate. After 20 hours, the medium was changed by fresh E medium with indicated concentration of drugs. After pre-treatment of drugs for 30 minutes, the medium was changed by fresh 200µl E medium with 100µM 2APB, 2mM camphor and indicated concentration of drugs for another 2 hours. Finally, cell viability and LDH releasing were measured as previous described. For Myr-AIP (TargetMol, USA) treatment, the pre-treatment time was extended to 3 hours to allow for enough penetration into cells.

Human skin single-cell sequence data analysis

The healthy human skin single sequence data was obtained and analyzed as previous described[3].

Statistics

Statistical and graphical analyses were performed with GraphPad Prism, version 9.0.0. Continuous variables were summarized by the mean and the SEM. Comparisons between 2 groups were performed using an unpaired, 2-tailed Student's t test, *P <= 0.05; **P <= 0.01; ***P <= 0.001; ****P <= 0.0001; ns, P>0.05, not significant. Some comparisons among more than 2 groups were performed by one-way ANOVA, *P <= 0.05; **P <= 0.01; ***P <= 0.001; ****P <= 0.0001; ns, P>0.05, not significant.

Supplementary References

1. Song, Z. *et al.* Hair Loss Caused by Gain-Of-Function Mutant TRPV3 Is Associated with Premature Differentiation of Follicular Keratinocytes. *J. Invest. Dermatol.* 1–11 (2021).

2. Moqrich, A. *et al.* Impaired thermosensation in mice lacking TRPV3, a heat and

camphor sensor in the skin. *Science* **307**, 1468–1472 (2005).

3. Xu, Z. *et al.* Anatomically distinct fibroblast subsets determine skin autoimmune patterns. *Nature* **601**, 118–124 (2022).

4. Xie, Y. *et al.* Hair shaft miniaturization causes stem cell depletion through mechanosensory signals mediated by a Piezo1-calcium-TNF-α axis. *Cell Stem Cell* **29**, 70-85.e6 (2022).

5. Qi, H. *et al.* Inhibition of temperature-sensitive TRPV3 channel by two natural isochlorogenic acid isomers for alleviation of dermatitis and chronic pruritus. *Acta Pharm Sin B* **12**, 723–734 (2022).

6. Tran, M. P. *et al.* Evolutionary loss of foot muscle during development with characteristics of atrophy and no evidence of cell death. *Elife* **8**, (2019).
